# *Pleione*: A tool for statistical and multi-objective calibration of Rule-based models

**DOI:** 10.1038/s41598-019-51546-6

**Published:** 2019-10-22

**Authors:** Rodrigo Santibáñez, Daniel Garrido, Alberto J. M. Martin

**Affiliations:** 1 Network Biology Lab, Centro de Genómica y Bioinformática, Facultad de Ciencias, Universidad Mayor, Santiago, 8580745 Chile; 2 Department of Chemical and Bioprocess Engineering, School of Engineering, Pontificia Universidad Católica de Chile, Santiago, 7820436 Chile

**Keywords:** Computer modelling, Software

## Abstract

Mathematical models based on Ordinary Differential Equations (ODEs) are frequently used to describe and simulate biological systems. Nevertheless, such models are often difficult to understand. Unlike ODE models, Rule-Based Models (RBMs) utilise formal language to describe reactions as a cumulative number of statements that are easier to understand and correct. They are also gaining popularity because of their conciseness and simulation flexibility. However, RBMs generally lack tools to perform further analysis that requires simulation. This situation arises because exact and approximate simulations are computationally intensive. Translating RBMs into ODEs is commonly used to reduce simulation time, but this technique may be prohibitive due to combinatorial explosion. Here, we present the software called *Pleione* to calibrate RBMs. Parameter calibration is essential given the incomplete experimental determination of reaction rates and the goal of using models to reproduce experimental data. The software distributes stochastic simulations and calculations and incorporates equivalence tests to determine the fitness of RBMs compared with data. The primary features of *Pleione* were thoroughly tested on a model of gene regulation in *Escherichia coli*. *Pleione* yielded satisfactory results regarding calculation time and error reduction for multiple simulators, models, parameter search strategies, and computing infrastructures.

## Introduction

Systems biology studies the behaviour of biological systems by determining and quantifying all of the molecular interactions that characterise them^1^. This area of science relies on different experimental, mathematical, and computational tools to address system generalities such as robustness and specific details such as bi-stability^2,3^. Notably, these computational approaches can be classified into two primary types: those that aim to determine cell component interactions (e.g., methods to infer Gene Regulatory Networks GRNs, from expression data^4^) and those used to study the dynamical properties that define such systems^1,2^.

In the post-genomic era, elucidating gene regulation remains one of the primary challenges of systems biology^5^. This information is highly relevant to understanding metabolism^6,7^, cell responses^8,9^, and cell-to-cell interactions^10,11^ and for developing industrial applications of microrganisms^12^. The increasing availability of omics data has facilitated modelling of biological systems with the goal of understanding and predicting their behaviour^13^. Frequently modelled experimental data include genomics, transcriptomics, proteomics, and metabolomics^14^. These datasets can be modelled in single-omics or integrated, multi-omics representations of cell behaviour^15^. Historically, Ordinary Differential Equations (ODEs)-based models have been extensively used for modelling biological systems^14^. Nowadays, Rule-Based Models (RBMs) are gaining popularity because of their advantages over their ODE counterparts^16–18^. For example, RBMs are best suited to modelling large systems that may be composed of millions of different types of components and transformations^17,18^. To simulate RBMs, most of the available tools use the Gillespie’s Stochastic Simulation Algorithm (SSA), a method to retrieve an exact numerical solution from a Chemical Master Equation^19^. The SSA and its derivatives are implemented in RBM simulators such as KaSim^17^, BioNetGen (BNG)^20,21^, and others^22–26^. Unfortunately, tools to perform calibration and determine parameter uncertainty of RBMs are generally lacking; they are available only for BioNetGen Language (BNGL) and Systems Biology Markup Language (SBML) models^27,28^. Without proper calibration, analysis of model perturbations and predictions beyond experimental data are impossible.

One significant difficulty of modelling biological systems is robustly estimating parameter values. This calibration procedure is especially relevant considering that the purpose of models is to reproduce observed data or phenomena^29^. Notably, this problem is computationally expensive in the case of RBMs, where conventional approaches rely on an equivalent ODE model to reduce the calculation time^30,31^. In general, multiple simulations of an RBM converge on a numerical solution to its ODE counterpart. However, both modelling frameworks entail different assumptions^16^. As a calibration example, Kozer *et al*.^30^ employed BNG to simulate multiple RBMs that differed only in parameter values, solving each model in a deterministic fashion with the CVODE software^32^. In contrast to Kozer *et al*.^30^, Aguilera *et al*.^31^ developed and performed a calibration of a stochastic model employing first-deterministic simulations. These authors argued that if a deterministic stationary state closely matches the modes of the experimental data, the employed parameters are good candidates for fitting stochastic simulations to the same data^31^. However, their approach fails if the stationary state is remarkably different from the simulated pseudo-stationary state of stochastic simulations, as was argued by Halh & Kremling^33^, or in situations where a deterministic simulation is not possible. For instance, KaSim^17^ does not provide an ODE solver and while KaDE^34^ can export a *kappa* model to ODEs in a variety of compatible software versions, the size of the generated model is affected by combinatorial complexity, the explosion in the number of ODEs due to the numerous interactions and modifications modelled^25^. For example, the EGFR/ERK pathway model contains 70 rules that are equivalent to approximately 10^23^ ODEs^17^. Despite the availability of robust methods to calibrate and analyse ODE models, using these methods to calibrate RBMs may disregard the stochastic behaviour of a system and accordingly result in a loss of useful information. To circumvent these problems, Thomas *et al*.^27^ developed BioNetFit (BNF) and recently Mitra *et al*.^28^ developed PyBioNetFit (pyBNF). Both of these tools harness computational load schedulers to parallelise simulations and cut down on the time necessary to calibrate an RBM. Although BNF (and pyBNF) address the need for a calibration tool, they support only RBMs written in BNGL and SBML^20^ and rely on algebraic equations to compare experimental data and simulation that may be of special concern depending on the nature of the modelled phenomena.

In this study, we present and describe the features of Pleione, an open resource to calibrate RBMs. *Pleione* encodes a Genetic Algorithm (GA), a robust and general methodology that searches the parameter space with operations that select, recombine, and mutate models with increased fitness^35^. *Pleione* was developed to perform three primary tasks: calibrate RBMs regardless of their underlying formal language, statistically assess models against experimental data, and distribute calculations with minimal user intervention. *Pleione* supports BNG2^20^, NFsim^23^, KaSim^17,21^, and PISKaS^36^ to perform simulations of RBMs either in BNG or *kappa* language. SBML models can be transformed with BNG into BNGL models^20^ or with PySB and exported to a myriad of formats^18^. Furthermore, *Pleione* can evaluate models employing unique or combined fitness functions^37^ referred to hereafter as single-objective GA (SOGA) or multi-objective GA (MOGA). Additionally, it incorporates parametric and non-parametric equivalence tests such as the two one-sided *t*-tests^38^, the Double Mann-Whitney U-test^39^, and the Wellek’s test^40,41^ as measurements of the fit between the experimental data and the stochastic simulations. *Pleione* can accordingly determine significant equivalences between experimental and simulated data. Lastly, we parallelised calculations employing the SLURM software, the de-facto standard for high-performance computing infrastructure. We also support parallelised calculations without SLURM using the python *multiprocessing* package.

We tested *Pleione* in a variety of settings and report its behaviour. We employed multiple search strategies with algebraic functions to calibrate 79 free parameters of the core GRN model of *E*. *coli*^36^. We also report the uncertainty in parameter values using jackknife and bootstrap procedures. Subsequently, we calibrated the Aguilera’s simple model of gene regulation^31^ employing the equivalence tests alone and in combination with a second fitness function. Finally, we provide a comparison of the developed method against BNF^27^, and we perform a calibration of an example RBM with six free parameters.

## Results and Discussion

### Single-objective genetic algorithm

#### Algebraic fitness functions

*Pleione* calculates nine algebraic equations commonly used as fitness functions (see the Methods section for the definition of these equations). We provide all fitness functions in a single file and separately from the *Pleione* main code for three reasons. First, the supported stochastic simulators report their results in different formats. Second, the separation enables transparent parallelisation of fitness calculations and its utilisation to validate models with new or independent data not used to calibrate the model. And third, the separation allows a straightforward and easy way to add new fitness functions following specific guidelines (https://pleione.readthedocs.io) and to give support to new deterministic or stochastic simulators.

**Figure 1:**
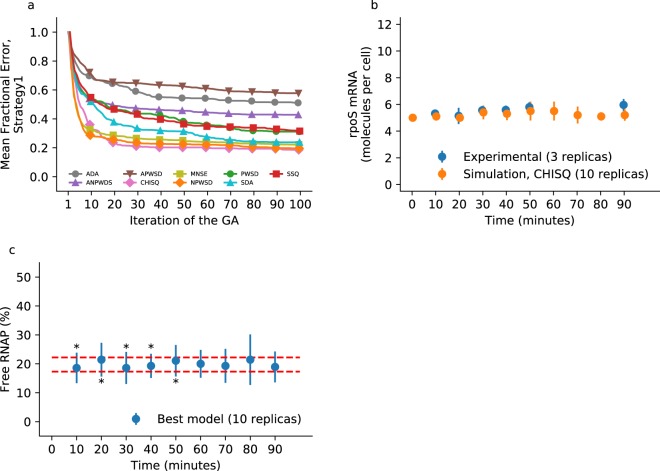
Parameter calibration using algebraic fitness functions. The RBM representing the core GRN of *E*. *coli* was calibrated against transcriptome data and evaluated algebraically. (**a**) Error convergence. The traces correspond to the mean of the 10 best models (the elite population) per iteration, normalised by the mean error at the first iteration (fractional error). (**b**) Comparison of the best model. The 10 simulations used to evaluate the best model at the end of the GA employing the chi-squared fitness function are plotted along with experimental data for the *rpoS* mRNA. The symbols correspond to the mean and one standard deviation. Data were plotted purposely with an offset to prevent the error bars from overlapping. (**c**) Independent validation. The calibrated model predicts free RNAP in a pseudo-stationary state in the 17–22% range; stars denote where a one-sample *t*-test concludes the model simulates a larger value (at 10 minutes, p-value ≈ 0.041; at 30 minutes, p-value ≈ 0.049; at 40 minutes, p-value ≈ 0.036) and a smaller value (at 20 minutes, p-value ≈ 0.038; at 50 minutes, p-value ≈ 0.040) compared with the 17–22% interval.

To test *Pleione*, we employed an elitist GA (see the Methods section, strategy 1) and transcriptomic data from Jozefczuk *et al*.^8^ to calibrate the core GRN model^36^. Jozefczuk *et al*. used a variety of conditions to evaluate the changes in mRNA expression using microarrays. We selected cold stress to exemplify the use of *Pleione*, although any experimental procedure that determines the abundance of mRNA molecules is suitable for matching with the model utilised in this work. We used *Pleione* with the same seed for the random number generator so all GAs started from the same population of models. The resulting error convergence for each fitness function is shown in Fig. 1A for the ten best models. To provide a fair comparison between each of the fitness functions, we report the fractional error that corresponds to the averaged error normalised by the average error at the first iteration for the elite of models. This procedure makes it possible to determine the fitness function characterised by the largest error reduction compared with its first iteration. For the tested model, calibration strategy, and fitness functions, the squared difference of averages (SDA, Eq. 1), the normalised pair-wise squared deviation (NPWSD, 5), the chi-square error (CHISQ, Eq. 8), and the mean normalised standard error (MNSE, Eq. 9) exhibited the best performances. These four fitness functions reduced the average error for the elite of models to nearly 20% of their original value (Fig. 1A).

Overall, we obtained good agreement between most of the experimental mRNA observations compared with their simulated values. Figure 1B shows the dynamics of the *rpoS* mRNA for the best-fit model based on CHISQ, and for all ten simulated mRNAs dynamics in Supplementary Fig. S1. The remarkably poor fit for *rpoA*, *rpoB*, and *rpoD* mRNAs (Supplementary Figs. S1C,D,F) may be explained by the lack of negative regulatory mechanisms in the model. There is a second regulatory layer in *E*. *coli* composed of antagonist proteins of sigma factor activity and a third layer that includes the antagonists of sigma-factor antagonists^36^. Incorporating those regulatory proteins into the model would benefit the calibration of mRNA responses that exhibit increased degradation and recovery, or vice versa. For instance, the *fecI* mRNA is rapidly synthesised and then degraded throughout the cold stress experiment (see Supplementary Fig. S1A), however, the model predicted positive net synthesis. Moreover, the model that we used as an example has constant levels of proteins. Extending the model beyond gene regulation to incorporate protein translation, degradation, and metabolism would increase the repertoire of responses and regulatory mechanisms that are active in a given condition. Finally, the model represents a small portion of the complete *E*. *coli* GRN and ignores all transcription factors and small regulatory RNAs with known function^42,43^ that directly or indirectly affect the dynamics of the considered mRNA. For instance, the Rsd protein accumulates throughout exponential growth and sequesters the RpoD protein, enabling higher activity of the sigma factor RpoS that drives gene expression in the stationary growth phase^44^. Another example is the *rpoH* mRNA that responds to high temperatures by restructuring its folding and increasing its translation rate^45^.

Complementary to the results, we also estimated the uncertainty on parameter values using leave-one-out jackknife and bootstrap resampling (Supplementary Tables S1 and S2). Furthermore, we provide the predicted values for all mRNAs, free and bound proteins, and protein complexes (40 variables) in Supplementary Video S1 (before calibration) and Video S2 (after calibration). The videos show that the model simulates a fast regime dominated by the formation of protein complexes during the first minute and then a slow regime dominated by the synthesis and degradation of mRNA later on. Small multiple snapshots of the model dynamics at 0, 1, 10, and 90 minutes are shown in Supplementary Fig. S2. To validate with independent data, we compared the free fraction of RNA Polymerase (RNAP) with that quantified experimentally by Patrick *et al*.^46^. The free RNAP simulated fraction was in close agreement with the reported value. In this case, the best model selected by the CHISQ function predicted a free RNAP fraction in the 17–22% range (Fig. 1C); an ANOVA test revealed no statistical difference among the nine simulated time points (F(8,81) ≈ 0.4185, p-value ≈ 0.9067). For comparison, Patrick *et al*. showed that the free fraction of RNAP in *E*. *coli* depends on the growth rate, and they reported a free fraction of 28% in fast-growing cells^46^.

#### Iterative equivalence tests as fitness functions

Statistical tests are commonly used to determine significant differences between treatments. Usually, researchers use parametric tests such as the paired and unpaired *t*-tests or their non-parametric counterparts, the Wilcoxon rank-sum and the Mann-Whitney U-test, to determine if responses are different^47^. However, the applicability of those statistical tests for calibrating models simulated stochastically is questioned in the base of their null hypotheses. While common statistical tests determine whether two particular values such as the mean or variance from two distribution are statistically different, equivalence tests determine opposite hypotheses. Equivalence tests aim to determine the significance of the extent to which two distributions can differ and also be equivalent for practical purposes^39^.

We incorporated three equivalence test in *Pleione*: the parametric two one-sided *t*-tests^38^ and the non-parametric Double Mann-Whitney U-test^39^ and Wellek’s test^40,41^. The two one-sided *t*-tests and the Double Mann-Whitney U-test are straightforward implementations of the t-test and U-test employed to determine whether a distribution shifted to the left and the right is now smaller and greater respectively compared to the other distribution. If one of the shifted distributions lie outside the predefined equivalence range, the equivalence test cannot reject its null hypothesis. On the other hand, Wellek’s test determines whether the probability of one random variable being greater than another is between a (small) range centred at 50%^40^. *Pleione* calculates the selected test for each time point and variable, a procedure conducted to build a rejection matrix. Then, the iterative equivalence test is defined as the sum of the non-rejected equivalence tests (i.e., tests that do not conclude the distributions are equivalent). A characteristic of the above definition is that the iterative equivalence tests are discrete functions with known limits: A perfect model has a score equal to zero, and a completely wrong model score equals the number of variables times the number of experimental time points. For instance, if the experimental setup measured ten variables for ten time points, the maximum score is 100. Additionally, defined in a way that counts how many failed tests were, the iterative equivalence tests are fitness functions suitable to be minimised, therefore useful to improve stochastic models through an evolutionary algorithm or similar procedure.

**Figure 2:**
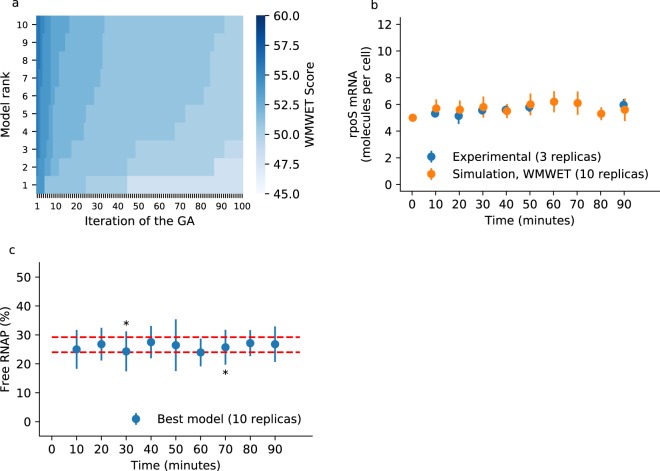
Parameter calibration using the non-parametric Wellek’s equivalence test. The RBM representing the core GRN of *E*. *coli* was calibrated against transcriptome data and evaluated algebraically. (**a**) Error convergence. The heat map correspond to the value of the 10 best models (the elite population) per iteration. (**b**) Comparison of the best model. The 10 simulations used to evaluate the best model at the end of the GA are plotted along with experimental data for the *rpoS* mRNA. The symbols correspond to the mean and one standard deviation. Data were plotted purposely with an offset to prevent the error bars from overlapping. (**c**) Independent validation. The calibrated model predicts free RNAP in a pseudo-stationary state in the 24–29% range; stars denote where a one-sample *t*-test concludes the model simulates a larger value (at 30 minutes, p-value ≈ 0.039) and a smaller value (at 70 minutes, p-value ≈ 0.037) compared with the 24–29% interval.

To determine the applicability of these equivalence tests, we used Aguilera *et al*.’s simple model of gene regulation^31^. These authors employed the model with known parameters to test their calibration method. Thanks to these known or “true” parameters, we constructed large batches of synthetic data. We randomly selected ten replications and calibrated the model using strategy 1. In general, the GA recovered the four “true” parameters. In the case of the two one-sided *t*-tests, the per cent error between the found and “true” parameters was no greater than 13.5% (31.2% cumulated per cent error for the four parameters) when using one standard deviation as symmetric equivalence range. Similarly, employing the Double Mann-Whitney U-test, the GA recovered all parameters reasonably well, with a cumulated per cent error close to 40%. Finally, employing the Wellek’s test with equivalence probabilities in the range of 19–76% (as similarly done in Wellek^40^) yielded two parameters with per cent errors less than 2.0%. However, the other two parameters were approximately 88% the value of the “true” parameters. All of the results are listed in Table 1. Next, we will show that combining the equivalence test with other search strategies and fitness functions greatly improved the calibration of the considered model. Also, results for the core GRN model calibrated with the Wellek’s test are shown in Fig. 2 and Supplementary Fig. S1. As depicted in Fig. 2A, calibration with the Wellek’s test yielded a low reduction in error, despite good agreement for *rpoS* mRNA (Fig. 2B) and free RNAP (Fig. 2C).

**Table 1:** Results of employing each equivalence test for the calibration of the Aguilera’s simple model^31^.

	Fitness function	Score	Parameters				Per cent error				Cumulative error
			r1_v	r2_k1	r3_k1	r4_k1	r1_v	r2_k1	r3_k1	r4_k1	
strategy1	TOST	4	5,672	0,034	0,097	0,029	13,43%	12,60%	−2,92%	−2,26%	31,21%
	DUT	3	5,672	0,034	0,094	0,028	13,43%	12,60%	−6,30%	−6,81%	39,14%
	WMWET	5	9,355	0,056	0,099	0,029	87,10%	87,77%	−1,47%	−1,71%	178,05%
strategy2	TOST	3	5,264	0,031	0,092	0,026	5,28%	3,22%	−7,79%	−12,49%	28,77%
	DUT	5	5,616	0,033	0,092	0,028	12,32%	10,99%	−7,79%	−6,93%	38,02%
	WMWET	4	5,275	0,032	0,094	0,028	5,49%	6,53%	−5,98%	−6,77%	24,77%
strategy3	TOST	5	8,493	0,050	0,216	0,015	69,85%	68,30%	116,40%	−49,29%	303,85%
	DUT	6	7,242	0,046	0,236	0,029	44,83%	52,36%	136,36%	−3,99%	237,54%
	WMWET	6	5,792	0,035	0,297	0,017	15,84%	17,43%	196,97%	−43,96%	274,20%
strategy3	TOST + SDA	8	5,065	0,032	0,099	0,029	1,29%	8,30%	−1,25%	−3,94%	14,78%
	DUT + SDA	7	6,117	0,037	0,091	0,027	22,34%	22,58%	−9,11%	−11,66%	65,70%
	WMWET + SDA	4	4,829	0,028	0,097	0,029	−3,42%	−6,89%	−3,46%	−3,71%	17,48%

Each strategy and fitness function (see the Methods section) was run once and the table shows the parameters of the best model at the end of the calibration. A comparison is shown for the percentual error compared to the “true” value of the parameters (r1_v = 5, r2_k1 = 0.03, r3_k1 = 0.1, r4_k1 = 0.03) and the cumulative error of all parameters.

### Multiple-objective genetic algorithm

Discrepancies between fitness functions can be resolved using multi-objective calibration. We present the fitness calculated by one of each fitness functions included in *Pleione* for the core GRN model (Supplementary Fig. S3). For nearly all of the fitness functions, the model with the lowest error was noted with the employed objective function as well with other fitness functions. This result reveals the strong correlation between the employed metrics (see Supplementary Fig. S4). The included fitness functions accordingly consider and measure the variability of data and simulations in different ways and justify the developed multi-objective capability in *Pleione*. There are multiple procedures for implementing such MOGAs, which were reviewed by Konak *et al*.^37^. We included the sum of individual rankings per fitness function (without weighting) to determine the contribution of each metric to the overall error. To exemplify the use of the multi-objective capability of *Pleione*, we selected multiple fitness functions based on the Spearman’s ρ correlation coefficient calculated between all fitness at the first iteration (see Supplementary Fig. S4). We could accordingly exclude fitness functions that would yield the same result after the calibration. In other words, the use of two highly correlated fitness functions is redundant. For instance, calibrations with the SDA (Eq. 1) and with the sum of squares function (SSQ, Eq. 7; see the Methods section) were highly correlated for the core GRN model; differences in the final error would be marginal if the user decided to use SDA instead of the other to calibrate the model. In the tested strategy 1, the fractional error for the elite of models employing SDA as fitness function was 23.8% while for SSQ was 31.6%.

**Figure 3:**
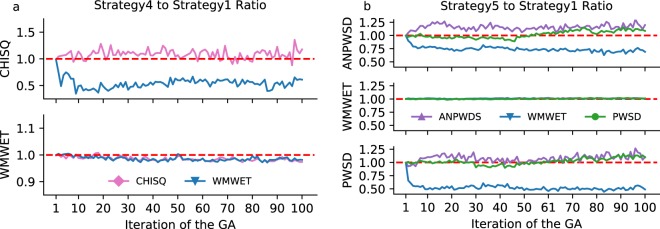
Comparison of MOGAs with their corresponding SOGAs. The RBM representing the core GRN of *E*. *coli* was calibrated against transcriptome data. (**a**) Strategy 4 mean ratio. The panels show the behaviour of the chi-squared (CHISQ, Eq. 8) and the Wellek’s test (WMWET) fitness functions in a multi-objective optimisation ratioed by the same error in the respective single-objective optimisation (minimising CHISQ, pink lines; or the Wellek’s test, blue lines). (**b**) Strategy 5 mean ratio. The panels show the behaviour of ANPWSD (Eq. 6), WMWET, and PWSD (Eq. 3) in a MOGA divided by their corresponding error in a SOGA (purple lines: minimising ANPWSD; blue lines: minimising WMWET; green lines: minimising PWSD). Values below 1.0 correspond to MOGA found an average error lower that SOGA. The legend indicates the objective function employed to calibrate the model.

We expect that combining fitness functions with low correlation among them will lead to better calibrations compared with using a single function. We found that the convergence behaviour of some fitness functions in a MOGA was comparable or better to those in a SOGA (see Supplementary Fig. S5). However, other fitness functions exhibited erratic behaviour consistent with a search strategy that attempts to satisfy multiple objectives at the same time. One remarkable example was the CHISQ (Eq. 8), where the error diverged compared with the single-objective optimisation in the tested configuration. The fit of *rpoS* mRNA is shown online in Supplementary Fig. S6 for two of the three tested calibration settings (see the Methods section, strategies 4 and 5). Comparisons between the MOGA and SOGA strategies are shown in Fig. 3 and Supplementary Fig. S7. Figures 3A,B, and S6 show the mean ratio between a fitness function in a MOGA for the same fitness function in a SOGA for strategies 4, 5, and 6, respectively. In general, utilizing a MOGA reduced the calibration error (i.e., the ratios were below unity), but modest or no improvement was observed for the Wellek’s test compared with the situation where the Wellek’s test was used as a single objective. Although, the primary rationale for using a MOGA is that the resulting model is more robust because it minimises multiple fitness functions simultaneously.

### Comparison with BioNetFit: The “example 6” model

**Figure 4:**
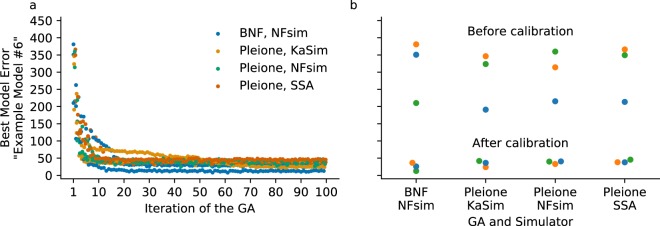
Comparison of calibration with BNF and *Pleione*. The “example 6” model from Thomas *et al*.^27^ was calibrated with synthetic data provided by the authors. Left. The traces correspond to the square root of the SDA (1) for the best model per iteration. Each GA was run three times with the same initial population of models for each stochastic simulator. Differences at the first iteration are due to the stochastic simulations of each model. Each model was simulated with the network-free simulators KaSim and NFsim and with the Gillespie’s SSA within the BNG software. Right. The dots indicate the error achieved at the last iteration (lower group) and the initial error at the first (blue, *seed* = 0), second (orange, *seed* = 2), and third independent calibration (green, *seed* = 2596283685).

Recently, Thomas *et al*.^27^ developed BNF intended for BNGL models, and Mitra *et al*.^28^ presented an improved version of Thomas’ software with more calibration algorithms and analysis (e.g., determination of the uncertainty of parameters values^48^). To perform a comparison between *Pleione* and BNF, we translated the “example 6” model (github.com/RuleWorld/BioNetFit) to PySB^18^ and then exported it to BNGL and *kappa*. The resulting models were calibrated with both BNF employing the network-free simulator NFsim and *Pleione* with the network-free simulators KaSim and NFsim, and the network-based Gillespie’s SSA within BNG2. Figure 4A shows the square root of the SDA (Eq. 1) for the best model achieved at each iteration. Our calibrations reveal that the error after calibrating the *kappa* “example 6” model is similar to the calibration with BNF. A comparison of the first and last iterations is shown in Fig. 4B, where each dot represents an independent calibration. The calibration with BNF yielded the smallest error. However, BNF only calibrates BNGL models; *Pleione* introduces the necessary methods to calibrate *kappa* RBMs. Moreover, we tested the equality of the independent runs using the Kruskal-Wallis H-test. We concluded that there was no significant difference between the lowest model errors at the last iteration (H(4) ≈ 4.128, p-value ≈ 0.248). This result further supports the notion that *Pleione* can perform as well as available tools for finding a candidate model. Notably, *Pleione* extends the use of GAs to a second RBM language as well to other stochastic simulators such as KaSim, PISKaS, and NFsim.

### Calibration of the core GRN model without using elitism

Multiple strategies have been developed to select individuals and generate new solutions from them with GAs^35^. To calibrate RBMs, BNF selects individuals to recombine from the entire population; *Pleione* choses individuals from an elite of models. Furthermore, BNF prohibits self-recombination by default while *Pleione* allows it. Moreover, another difference between the approaches pertains to the probability distribution used to select a model as a parent. In BNF, the selection is inversely proportional to the rank (see the BNF Manual^27^); *Pleione* selects individuals from a uniform distribution within the elite of models. To thoroughly analyse the developed method, we also calibrated the core GRN model with *Pleione* following an inverse rank strategy to select parents within the GA. This technique is referred to as strategy 2 (see the Methods section). For instance, from a population of 100 individuals, the first-ranked model has a probability of 19.28% of being selected as a parent; the second-best model has a probability of 9.64%.

**Figure 5:**
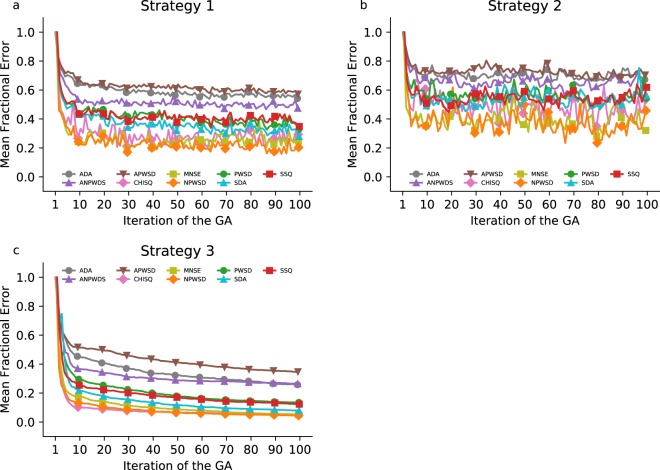
Calibration of the core GRN model with strategies 1, 2, and 3. The RBM representing the core GRN of *E*. *coli* was calibrated against transcriptome data and evaluated individually. (**a**–**c**) Error convergence in a single-objective calibration. The traces correspond to the mean of all models per iteration, normalised against the maximum value achieved at the first iteration (fractional error).

The calibration results of the core GRN model with all fitness functions are shown in Fig. 5A for the elitist GA (see the Methods section; strategy 1), in Fig. 5B for the non-elitist GA (strategy 2), and in Fig. 5C for strategy 3, the selection and recombination strategy most similar to BNF. Notably, the results revealed that the selection of parents implemented in *Pleione* (referred to as strategy 1) outperformed the selection of parents implemented in BNF (strategy 2) for the core GRN model (Fig. 5B). That is, the selection of individuals from an elite of models was more beneficial to find better solutions than selecting individuals from all models. These results must be specifically interpreted for the considered model in which the number of parameters is nearly 13 times larger than in the “example 6” model. Moreover, the parameter search benefits from other feature in BNF that recombines two individuals in multiple points, not just one as initially tested. We performed a calibration employing an inverse to the rank selection, a multiple crossing-over, and a mutation strategy within a ±10% range of the original parameter value (strategy 3). The results, shown in Fig. 5C, expose that the strategy used in BNF performed better for the considered model. For instance, the fractional error at the final iteration with the CHISQ error was roughly 5% of the original error for all models. That is nearly a six-fold improvement compared with strategy 1, where the fractional error was 29% for all models.

Complementary to the calibration of the core GRN model, we calibrated the simple model of gene regulation with each one of the equivalence tests using strategy 3. In the case of the Wellek’s test, the per cent error for each parameter was as low as 3.42% and as high as 6.89%, with a total per cent error of 17.48%, but only when combined with SDA (Eq. 1). In general, strategy 3 exhibited worst performance than strategy 1 at recovering the “true” parameters using equivalence tests. In the case of Wellek’s test, the total per cent error was increased from 178% (strategy 1) to 274% (strategy 3) and in all cases reduced employing a second fitness function (e.g. SDA, Eq. 1) for calibration employing strategy 3 (see the Methods section). All of the results are listed in Table 1.

## Conclusion

Parameter estimation is a common problem when developing predictive models in systems biology. In the case of stochastic simulations, the problem is magnified when data variability is disregarded using deterministic simulations to reduce simulation time or when fitness functions disregard simulation variability even when the model is simulated stochastically. Our method, *Pleione*, solved these two issues. *Pleione* takes less time to calibrate as the increasing availability of CPUs reduces the burden of multiple stochastic simulations, leveraging the need for deterministic simulations of RBMs. Parallelisation permits *Pleione* could use a sufficient number of stochastic simulations to be compared with experimental data. Regarding fitness functions, we propose using equivalence tests to determine similarity of data and simulations. Although we included parametric and non-parametric tests, the Wellek’s test makes it possible to render a confident assessment of the pertinence of a few stochastic simulations to reproduce a small number of experiment replications. Moreover, our approach provides support to *kappa* RBMs and can select models according to multiple metrics which overcomes the drawbacks of each fitness function.

One limitation of our method is the source of noise as *Pleione* does not currently consider experimental variability. Also, *Pleione* does not perform identifiability and parameter uncertainty determination on its own. We developed Alcyone (see the Methods section) to supplement this deficiency using jackknife and bootstrapping methods. However, Approximate Bayesian Computation (ABC, reviewed by Warne *et al*.^49^) has strengths in terms of identifiability and parameter uncertainty. ABC methods are preferred, but they take exceedingly long computational times as the discrepancy threshold is lower (as shown by Warne *et al*.^49^) and selecting the discrepancy metric may be non-trivial.

Features within *Pleione* are extensible. The incorporation of a noise model into simulated data, new fitness functions, and other statistical approaches like the ones introduced by Liu and Faeder^50^ and others^49^ are considered and will take advantage of parallelisation of simulations and calculations. Finally, *Pleione* is part of a larger project that will introduce common procedures to analyse RBM like swarm particle optimisation^51^ (already present in Mitra’s pyBNF), maximum likelihood optimisation^52^, and sensitivity analysis^53,54^ of parameter values, all based on parallelisation of stochastic simulations. We also expect that *Pleione* will facilitate broader adoption and development of RBMs to reproduce the structure and dynamics of complex biological systems.

## Methods

### Software implementation

*Pleione* is open software written in python3 and it is cross-platform compatible. The GA implemented is a simple iterative process of selection, recombination, and mutation of parameter values^35^. Before performing any optimisation, *Pleione* reads an RBM and identifies the free parameters in the model, i.e., user-selected variables that are going to be calibrated and their allowed search space. After building the first population, *Pleione* writes as many models as defined by the user with each parameter set; it then queues the simulation jobs using SLURM or the python *multiprocessing* API. After the first population is simulated, the models are ranked using one or more of the nine algebraic and two statistical fitness functions. Then, two selection strategies can be used. The first is a uniform probability selection that selects two parents from the best (“elite of”) models. The second strategy employs a distribution that is inversely proportional to the rank (and optional elitism). The latter is a selection strategy previously implemented in BNF^35^. The user can control crossover and select between single and multiple crossover points. Finally, each parameter value can be mutated, and a new value can be selected from a uniform or log-uniform distribution or multiplied by a random factor centred on the old parameter value. The simulation, selection, and mutation procedures are repeated until the number of iterations reaches the user-defined value. *Pleione*’s default is to perform elitism with multiple crossing points and parameter mutations from a uniform distribution. *Pleione* is able to perform calibrations employing BNG2^20^, NFsim^23^, KaSim^17,21^, and PISKaS^36^, regardless of differences in model configuration, command-line interface, or format of the reported simulation results. Other (stochastic) simulators can be incorporated into *Pleione* if they provide a command-line interface. *Pleione* is freely available at Python Package Index and Github (see the *Pleione* Manual for installation and user instructions at https://pleione.readthedocs.io).

### Fitness functions

#### Software implementation of iterative equivalence tests

Equivalence tests aim to determine the significance of the extent to which two distributions differ or are equivalent to practical purposes^39^. The tests determine the rejection of one of the two null hypotheses that the difference lies beyond the equivalence range. The first of these implemented tests were the “two one-sided *t*-tests”^38^ from the python *statsmodels* package with a predefined 5% significance level. The fitness function is selected by its acronym TOST (see the *Pleione* Manual for details at https://pleione.readthedocs.io). TOST shifts simulated values to the left and right and test whether the resulting distribution is statistically smaller or larger than unaltered data. Only when both unpaired *t*-tests reject their *null* hypotheses does TOST reject its own.

The second test, the Double Mann-Whitney U-test, is a straightforward adaptation of the Mann-Whitney U-test as mentioned by Cornell^39^ and discussed by Wellek^40^. The fitness function is selected by its acronym DUT (see the *Pleione* Manual for details at https://pleione.readthedocs.io). Like TOST, DUT shifts the simulated variables toward the left and the right and compares them with unaltered data using a one-sided U-test. The U-test determines whether a random variable is stochastically larger than another random variable^55^; it is the non-parametric counterpart of the unpaired *t*-test. To calculate the U-test, we first determined how many experimental values were smaller and greater than the shifted simulated values using Algorithm 1. Here, *exp* stands for an experiment replication; *shifted sim* stands for shifted stochastic simulations of an RBM. *Lower* and *upper* are the threshold limits. After comparing the experimental data and simulations, the observed difference *U*_*exp*_ and *U*_*sims*_ were compared against a critical value using Algorithm 2.

**Algorithm 1:**
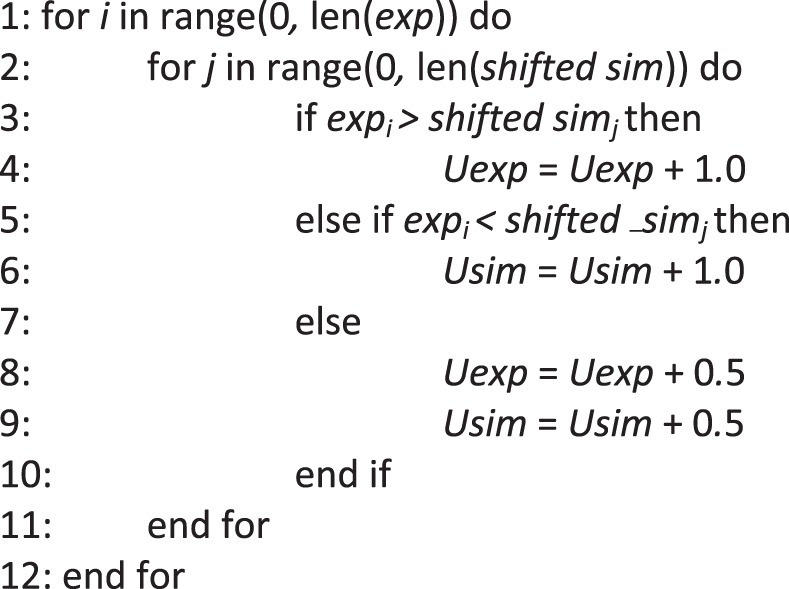
Count how many times experimental data is larger than shifted simulated values.

**Algorithm 2:**
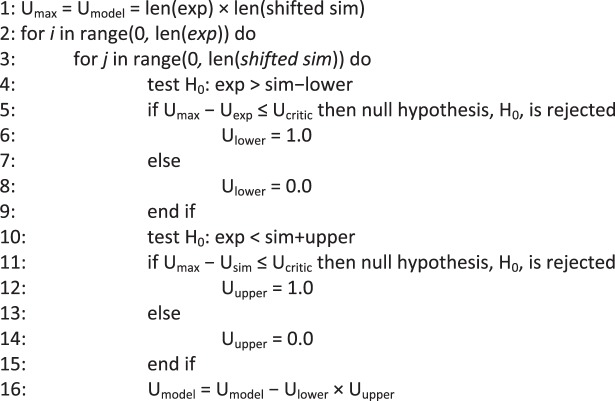
Double Mann-Whitney U-test. Determine if the difference is statistically significant for each variable.

The third equivalence test is the Wellek’s test^40,41^ that determines whether if the probability of the difference of two random variables lies within a (small) threshold around 50%. We implemented the mawi.R routine from the EQUIVNONINF R package (https://rdrr.io/cran/EQUIVNONINF/) in python. *Pleione* uses WMWET as an acronym for the fitness function.

All three equivalence tests were calculated for each variable and time point, and the fitness functions minimise the total sum of succesful equivalence tests substracted from the total number of performed tests (e.g. U_max_ in Algorithm 2).

#### Algebraic functions

*Pleione* includes nine algebraic fitness functions which are described below with their acronyms in parentheses. As noted previously, *exp* stands for an experiment replication, stands for a simulated value, $\bar{exp}$ refers to the average, and *σ*_*exp*_ is the standard deviation of experimental values.

- Squared Difference of two Averages (SDA):

1 $${(\frac{1}{m}\mathop{\sum }\limits_{i=1}^{m}ex{p}_{i}-\frac{1}{n}\mathop{\sum }\limits_{j=1}^{n}si{m}_{j})}^{2}$$

- Absolute value of the Difference of two Averages (ADA):

2 $$|\frac{1}{m}\mathop{\sum }\limits_{i=1}^{m}\,ex{p}_{i}-\frac{1}{n}\mathop{\sum \,}\limits_{j=1}^{n}si{m}_{j}|$$

- Pair-Wise Square Deviation (PWSD):
  3 $$\frac{1}{mn}\mathop{\sum \,}\limits_{i=1}^{m}\mathop{\sum }\limits_{j=1}^{n}\,{(ex{p}_{i}-si{m}_{j})}^{2}$$
- Absolute Pair-Wise Deviation (APWSD):
  4 $$\frac{1}{mn}\mathop{\sum \,}\limits_{i=1}^{m}\mathop{\sum }\limits_{j=1}^{n}\,|(ex{p}_{i}-si{m}_{j})|$$
- Normalised Pair-Wise Square Deviation (NPWSD):
  5 $$\frac{1}{mn}\mathop{\sum }\limits_{i=1}^{m}\,\mathop{\sum }\limits_{j=1}^{n}\,{(\frac{ex{p}_{i}-si{m}_{j}}{ex{p}_{i}})}^{2}$$
- Normalised Absolute Pair-Wise Deviation (ANPWSD):
  6 $$\frac{1}{mn}\mathop{\sum }\limits_{i=1}^{m}\,\mathop{\sum }\limits_{j=1}^{n}|(\frac{ex{p}_{i}-si{m}_{j}}{ex{p}_{i}})|$$
- Sum of Squares (SSQ)^27^:
  7 $$\mathop{\sum }\limits_{i=1}^{m}\,\mathop{\sum }\limits_{j=1}^{n}{(ex{p}_{i}-si{m}_{j})}^{2}$$
- Chi-Square (CHISQ)^27^:
  8 $$\mathop{\sum }\limits_{i=1}^{m}\,\mathop{\sum }\limits_{j=1}^{n}{(\frac{ex{p}_{i}-si{m}_{j}}{{\sigma }_{exp}})}^{2}$$
- Mean Normalised Square Error (MNSE)^27^:

9 $$\mathop{\sum }\limits_{i=1}^{m}\,\mathop{\sum }\limits_{j=1}^{n}{(\frac{ex{p}_{i}-si{m}_{j}}{\bar{exp}})}^{2}$$

### Models and experimental data

#### Calibration of transcriptional dynamics

To test *Pleione*, we employed a simple model of gene regulation^31^ and the core GRN model of *E*. *coli* that we published previously^36^. The former is a four-equation model developed and used by Aguilera *et al*.^31^ with known parameters to create a synthetic dataset. The equations describe the synthesis and degradation of an mRNA and its encoded protein. We randomly selected ten simulations using the “true” parameters and then set all rates as free parameters. The latter model resembles the GRN, protein-protein interactions, and genome architecture of *E*. *coli* K-12 (see Supplementary Fig. S8). The core GRN is composed of ten genes, of which seven encode sigma factors^56^. The other genes correspond to the α, β, and β’ subunits of the *E*. *coli* RNAP. This GRN was built from EcoCyc^43^ and complemented with literature data^56^. It resulted in a network of 30 positive regulations (see Supplementary Fig. S8). The experimental data used to calibrate the model correspond to microarrays of *E*. *coli* gene expression after stress performed by Jozefczuk *et al*.^8^ (GEO accession GSE20305). These data were processed as in Marbach *et al*.^4^, and the resulting values were assumed to represent the absolute quantity of mRNA per cell for each available time point after stress induction (see Supplementary File S1). We selected the binding and unbinding of each sigma factor to the core RNAP (14 parameters), the binding and unbinding of the seven RNAP holoenzymes to their cognate promoters (56 parameters), and the decay of RNA molecules (9 parameters) as free parameters.

We employed six different GA configurations, which are described below. All strategies included 100 models simulated ten times through 100 iterations. We repeated the calibration from the same initial population choosing the same random number generator seed to test the fitness functions included in *Pleione*. All of the simulations for the Aguilera’s simple model were done with BNG v2.2.6 (https://github.com/RuleWorld/bionetgen/releases/tag/BioNetGen-2.2.6-stable). For the core GRN model, the simulations were done with KaSim v4.0 (https://github.com/Kappa-Dev/KaSim/releases/tag/v4.0). The scripts to repeat the calibration are in Supplementary File S2. The differences between strategies to calibrate are as follows:

- Strategy 1, elitist GA: After each iteration, the ten best models were selected and left unaltered until the next iteration. From this elite of models, two parents were selected with a uniform probability and crossed in a single, random point. We allowed self-recombination of parents. Mutation of parameters resulted in a new value from the original range with a 30% probability. We did not repeat simulations from the elite population.
- Strategy 2, non-elitist GA. After each iteration, all models were subjected to selection with an inverse to the rank probability distribution. For instance, the model *i* with rank *r*_*i*_ had a probability of selection *p*_*i*_ equal to $1/({r}_{i}\mathop{\sum }\limits_{j=1}^{R}1/{r}_{j})$. After selecting two different parents, they were recombined in a single, random point. The mutation of parameters values was the same as in Strategy 1.
- Strategy 3. The same as Strategy 2, but the mutation of parameters yielded a new value with a 20% probability from a random factor within a ±10% interval centred at the old value. This strategy was applied using BNF.
- Strategies 4, 5, and 6 (MOGA). These strategies were similar to Strategy 1, but we selected the CHISQ (Eq. 8) and WMWET; the ANPWSD (Eq. 6), WMWET, and PWSD (Eq. 3); and the CHISQ, WMWET, SDA (Eq. 1), and NPWSD (Eq. 5) simultaneously as fitness functions for the rank models.

#### Comparison with BioNetFit

*Pleione* was also used to calibrate an equivalent model to “example 6” reported by Thomas *et al*.^27^ in *kappa* language. Although Thomas *et al*. published another two models, some of the BNGL syntax within them do not have a simple or equivalent expression in *kappa* language. The “example 6” is a toy model with synthetic experimental data that resemble the ligand-induced autophosphorylation of a receptor^27^. The model was rewritten employing PySB version 1.5.0^18^ and exported to *kappa* and BNGL. The latter was simulated with BNG2 and compared with the original model to discard syntax misinterpretation while rewriting the model. The *kappa* model was further modified to enable an equilibration step similar to that of the original model. To perform the comparison with BNF, both GAs were run employing strategy 3. The first population was drawn from a log-uniform distribution between 10^−2^ and 10^+2^. The same options were used when calibrating with BNF, and we selected equivalent objective functions to fit with BNF and *Pleione*. The simulators were BNG v2.2.6 https://github.com/RuleWorld/bionetgen/releases/tag/BioNetGen-2.2.6-stable and NFsim v1.12.1, which was compiled from source obtained at the BNF GitHub repository (https://github.com/PosnerLab/BioNetFit). BNF was obtained from https://github.com/RuleWorld/BioNetFit/releases/tag/v1.01.

### Complementary analysis and statistical tests

The core GRN model simulates the availability of RNAP, also referred to as the free RNAP fraction. To determine whether the simulated free fraction was in a pseudo-stationary state, we employed the one-way ANOVA test and the Kruskal-Wallis H-test, both from the python SciPy package^57^. Additionally, to determine the probability of a range of simulated free RNAP, we repeatedly employed the one-sample *t*-test with a 95% confidence level in a one-tail test; we adjusted the threshold of free RNAP fraction over the entire time interval until the *t*-test rejected the null hypothesis at least once. Finally, to determine which fitness functions to employ in a multi-objective GA, we calculated the Pearson’s, Spearman’s rank (*ρ*), and Kendall’s rank (*τ*) correlation coefficients, which were also implemented within the SciPy package^57^. Parameter uncertainty for the core GRN model was assessed using Alcyone https://github.com/networkbiolab/alcyone/tree/master/example employing the leave-one-out jackknife and the bootstrap resampling methods. The latter was performed with 20 GA runs, which enabled a maximum 90% confidence interval. Animations of the dynamics were prepared from 1000 simulations with KaSim. We plotted the average and one standard deviation for a total of 40 variables during the first minute at intervals of one second; the remaining simulated times had intervals of 0.1 minutes (i.e., 6 seconds).

## Supplementary information

File S1

Simulation before calibration

Simulation after simulation

Supplementary figures and tables

## Data Availability

All data generated during this study are included in this published article (and its Supplementary Information files).
