# Supplementary material for: Pleione: A tool for statistical and multi-objective calibration of Rule-based models
Source: Sci Rep. 2019 Oct 22;9:15104. doi: 10.1038/s41598-019-51546-6 (PMC6805871; doi:10.1038/s41598-019-51546-6)
Supplement: Supplementary file 4 — Supplementary figures and tables [file 41598_2019_51546_MOESM4_ESM.pdf]

Supplementary figures and tables for *Pleione*: A tool for statistical and multi-objective calibration of Rule-based models

Rodrigo Santibáñez[1,2], Daniel Garrido[2], and Alberto J.M. Martin[1,*]

[1]Network Biology Lab, Centro de Genómica y Bioinformática, Facultad de Ciencias, Universidad Mayor, Santiago, 8580745, Chile
[2]Department of Chemical and Bioprocess Engineering, School of Engineering, Pontificia Universidad Católica de Chile, Santiago, 7820436, Chile
[*]alberto.martin@umayor.cl

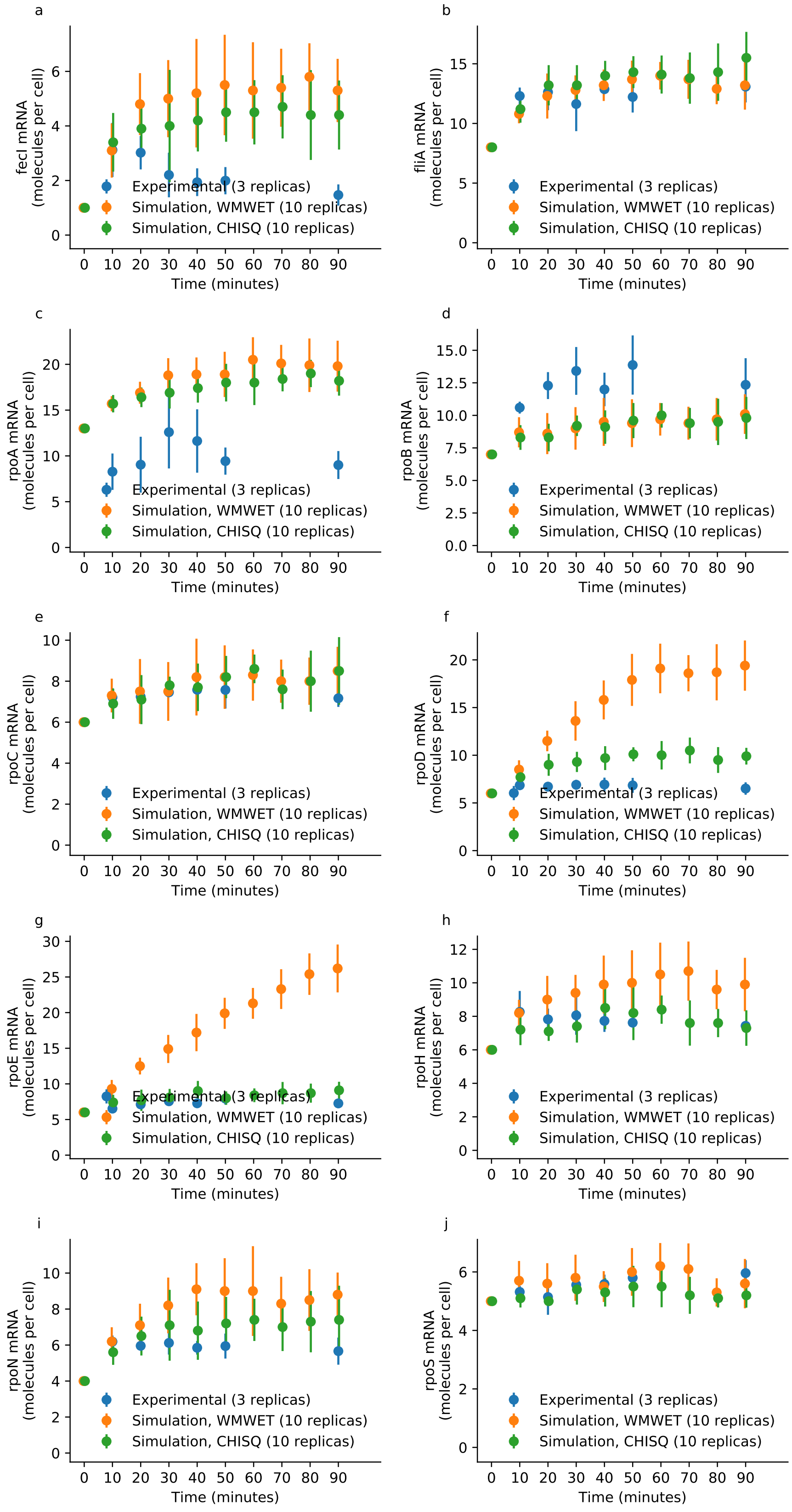

Figure S1: Simulation of the best parameter set after calibration of the Core GRN Model with the iterative Wellek's test (WMWET) and the chi-square (CHISQ) fitness functions employing Strategy1. The panels show one of the ten mRNA which dynamics were reproduced by the Core GRN Model at some extent. There are 4 main outcomes: good agreement for both metrics, like the case of rpoC and rpoS mRNA; good agreement with only one fitness functions, for instance the rpoB and rpoE mRNAs; and complete lack of fit with both, as is seen for the rpoD mRNA and others.

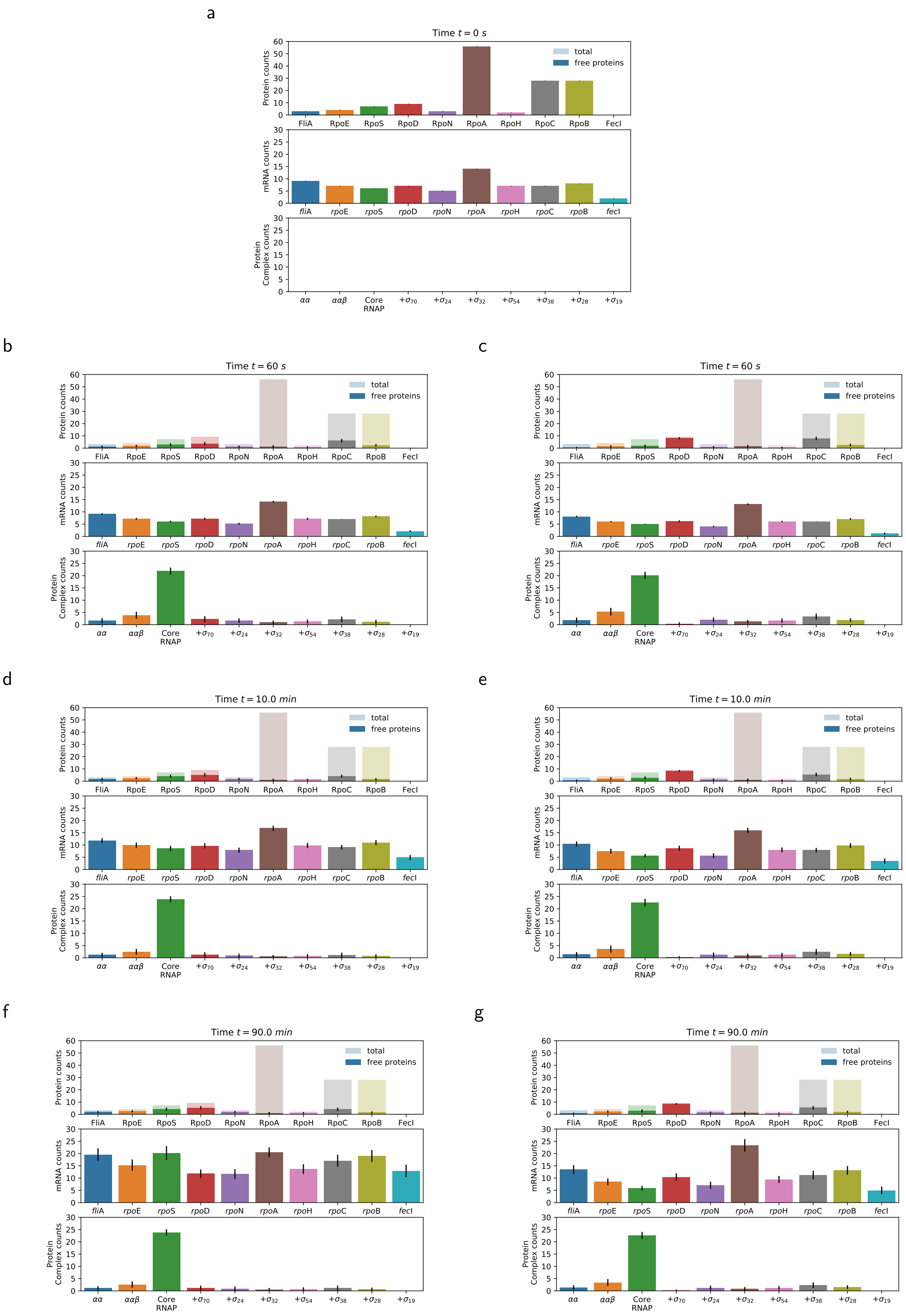

Figure S2: Small multiple depicting the initial condition (panel A) and how it evolved through the simulated time (1, 10, and final time 90 minutes) when model parameters before (panels B, D, and F) and after calibration (panels C, E, and G).

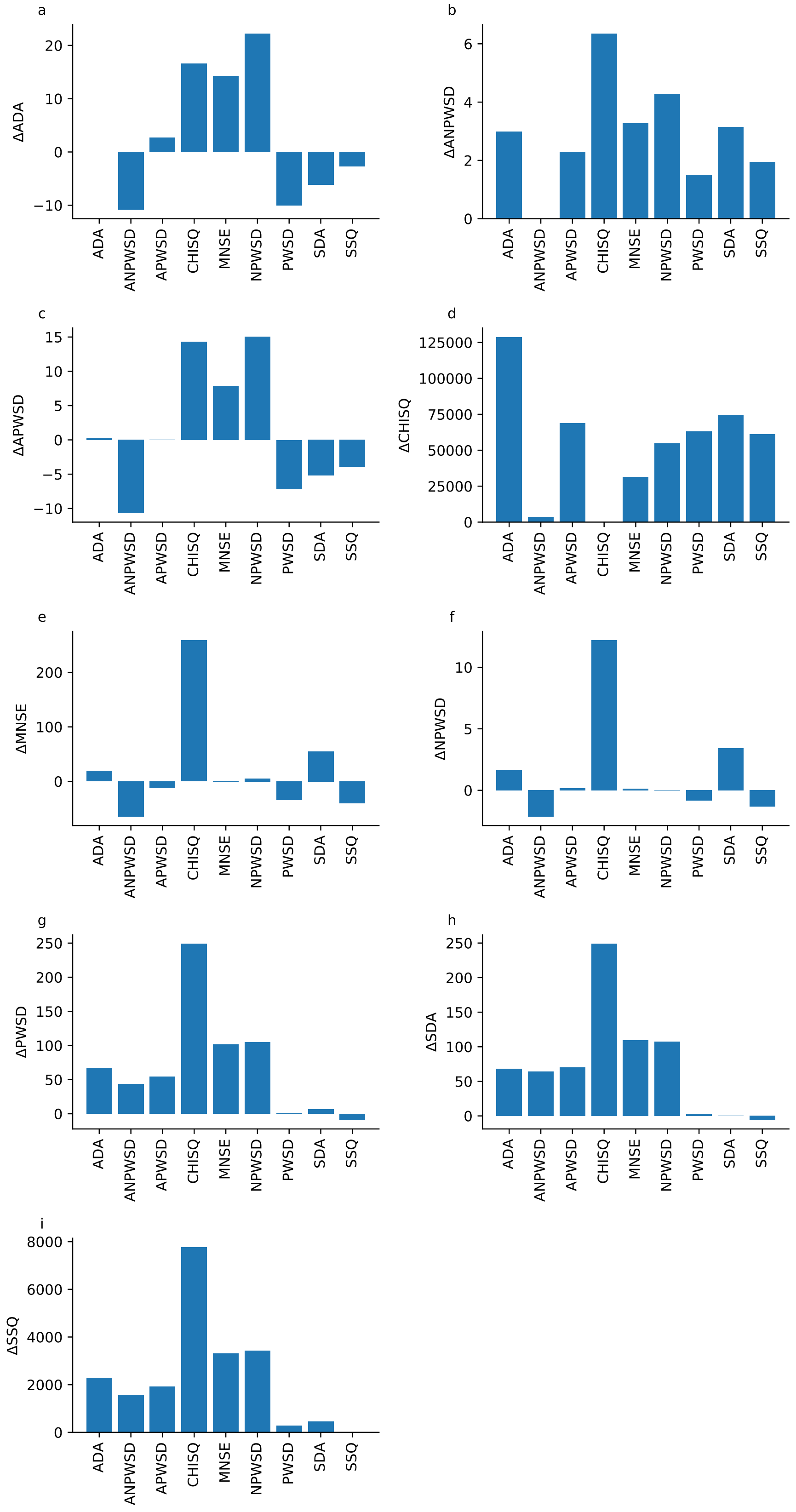

Figure S3: Difference for the error of the best models calibrated with Strategy1 at each single fitness function. The error determined for the first rank model at the end of the calibration is shown subtracted by the error found when the same fitness function was use as the objective function. Most of the fitness functions shows equal or lower capability to reduce other fitness errors, indicated by the positive difference. Interestingly, the absolute value of the difference of two averages (ADA, panel A) as well other fitness functions were minimized even further employing another function as the objective.

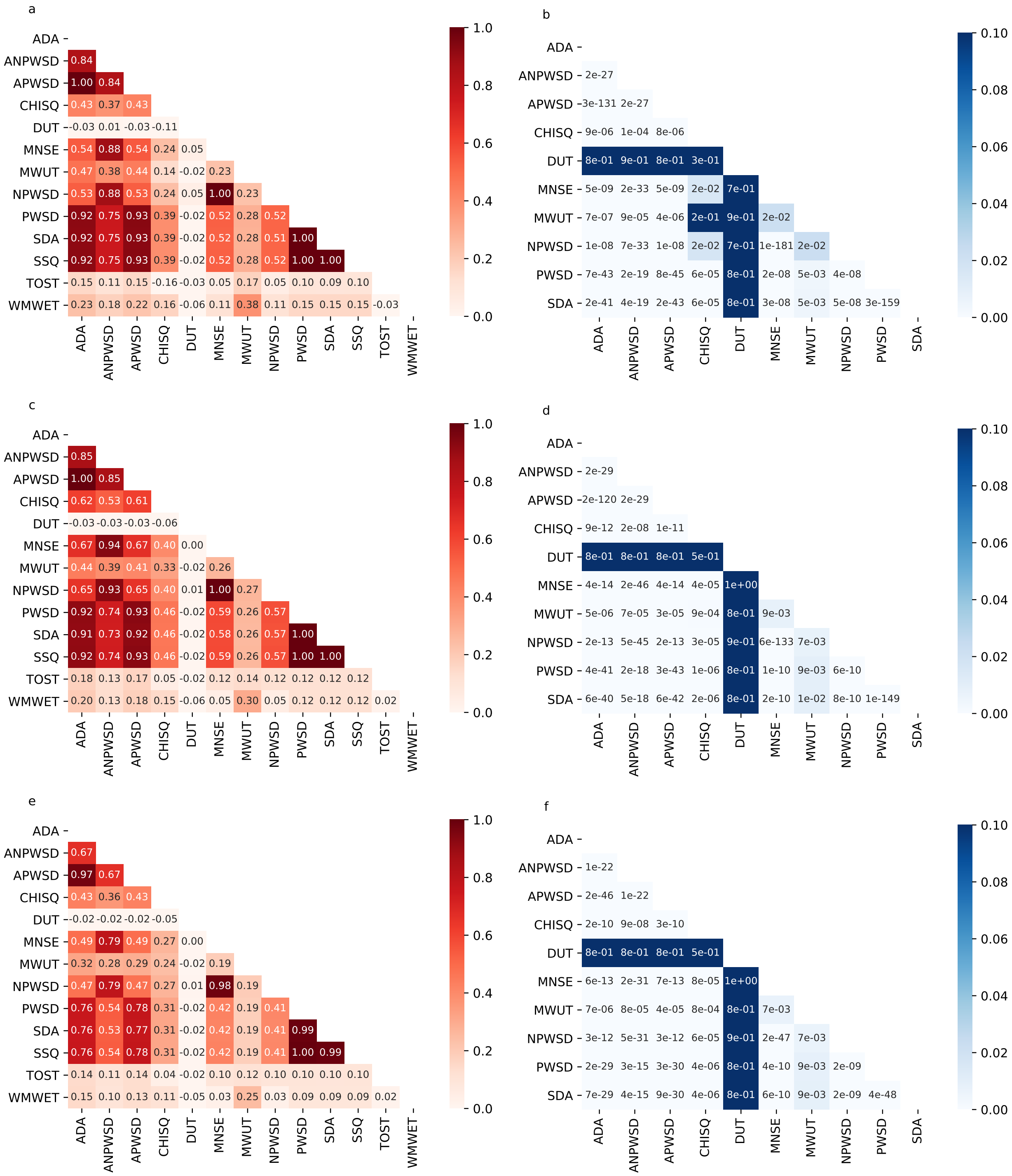

Figure S4: Correlation coefficients (left) and p-values (right) for the ten fitness functions included in *Pleione*. **A,B.** Pearson's correlation coefficient; **C,D.** Spearman ρ correlation coefficient; and **E,F.** Kendall τ correlation coefficient. All coefficients were calculated with the python SciPy package at the first iteration of 100 individuals to calibrate the Core GRN Model.

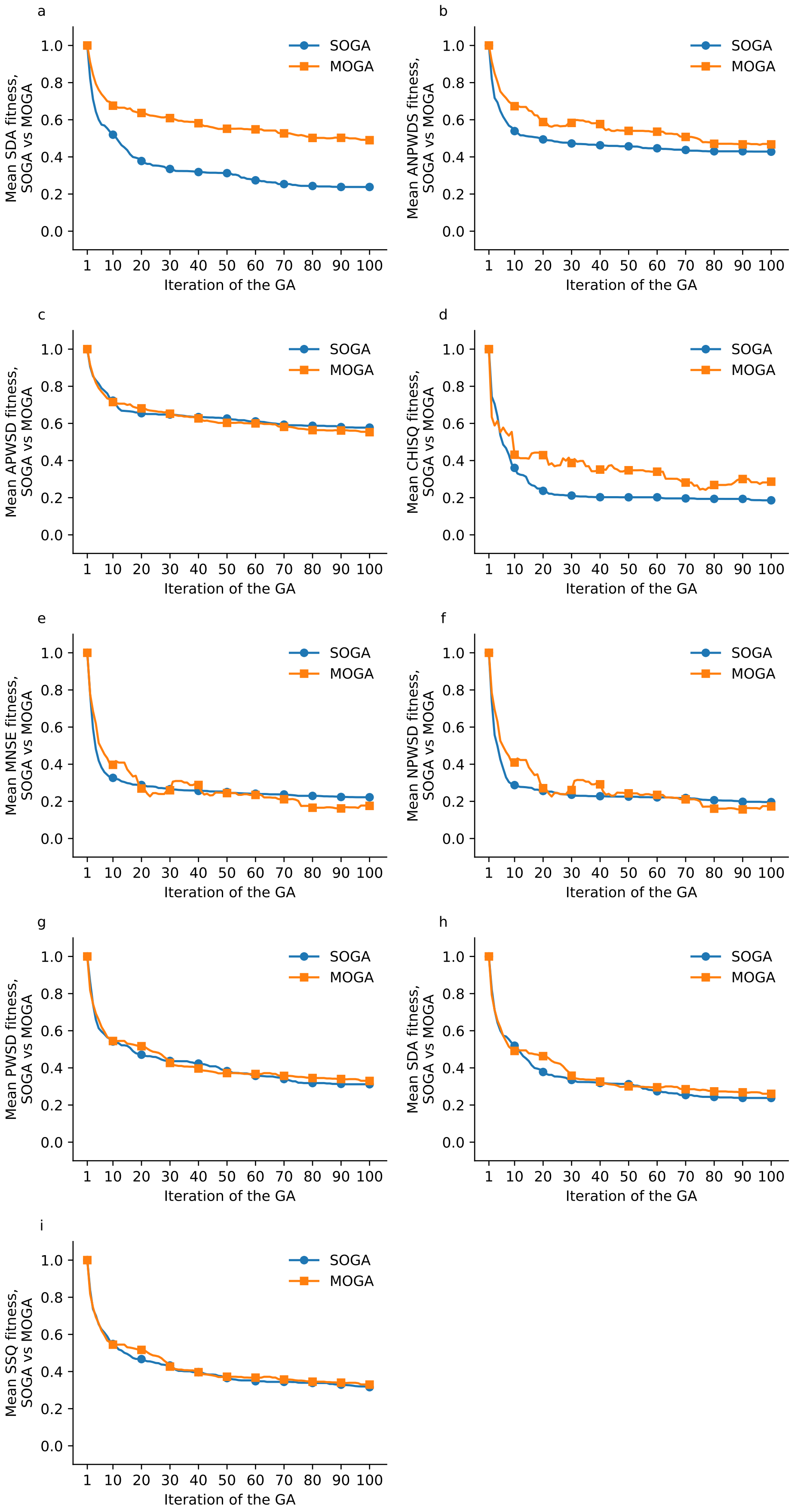

Figure S5: Fractional mean error convergence for a calibration employing Strategy1 (blue circles) and Strategy4 (orange squares). Each panel shows the convergence for a single fitness function while it was selected to calibrate the Core GRN Model, and comparatively with the same error while was selected simultaneously the ANPWSD (first row, right panel), the Wellek's test, and PWSD (fourth row, left panel) fitness functions.

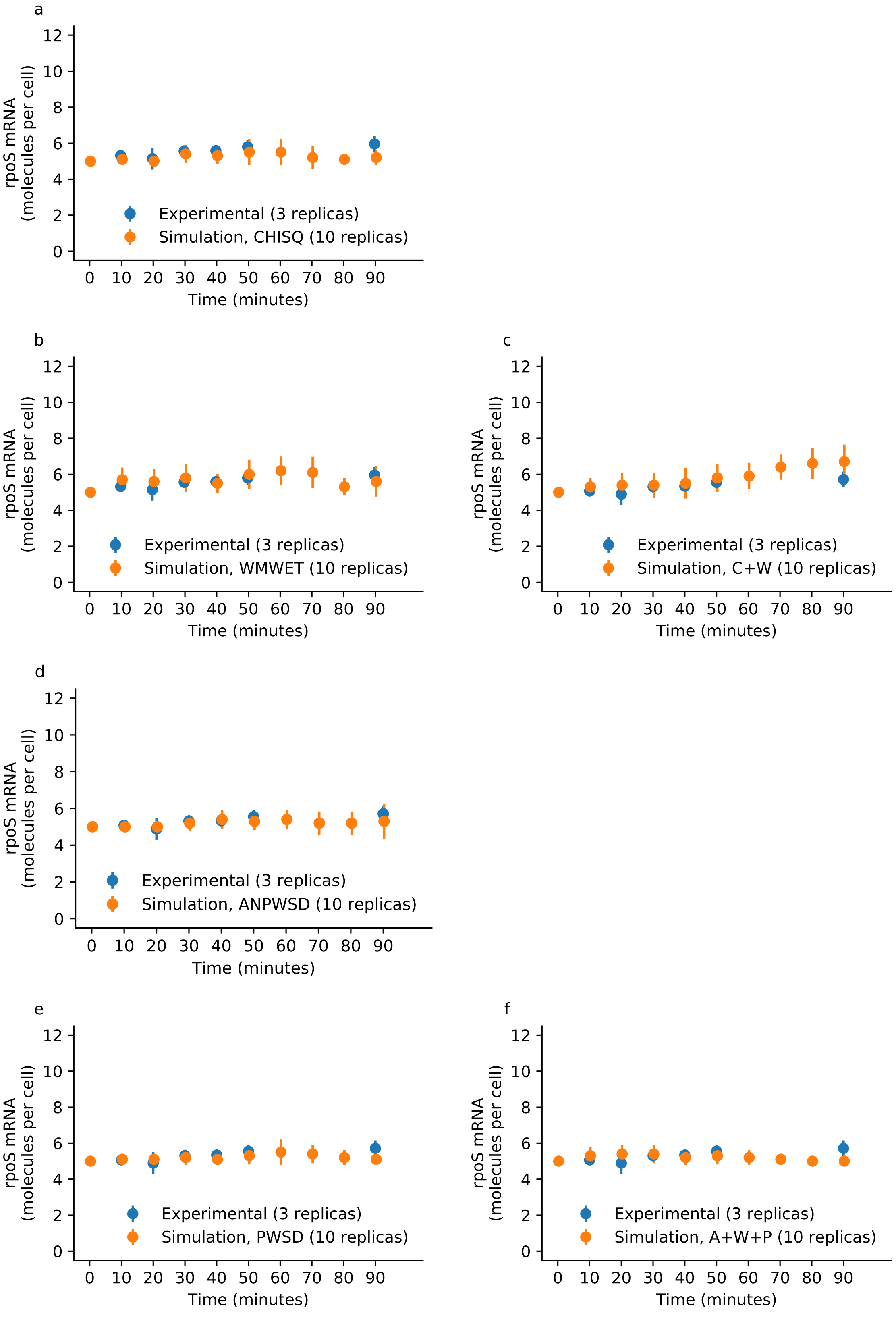

Figure S6: Best fits for the rpoS mRNA employing a single (left panels) and multiple fitness functions (right panels) simultaneously. The two Multi-Objective calibrations were performed employing the chi-square and the Wellek's test (panel C), and the other with the absolute pair-wise deviation, the Wellek's test, and the normalized absolute pair-wise deviation fitness functions (panel F). Differences are marginal between each calibration.

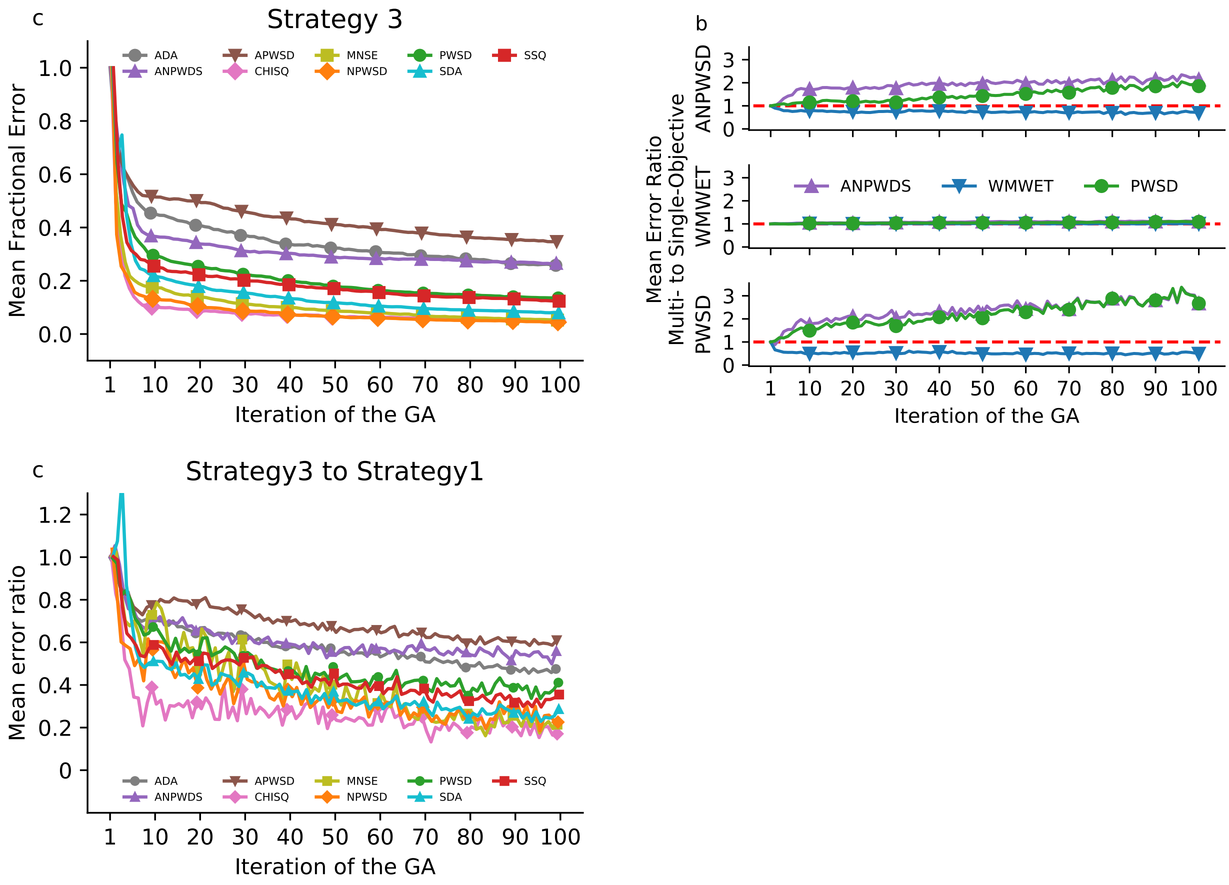

Figure S7: **Calibration with *Pleione* with the Strategy3.** The RBM representing the Core GRN of *E. coli* was calibrated against transcriptomic data and evaluated individually with all ten fitness functions. **A. Error convergence in a Single-Objective Calibration.** The traces correspond to the mean of all models per iteration, normalized against the maximum value achieved at the first iteration (fractional error) **B. Multi-Objective Performance.** The traces correspond to the ratio between a single fitness function in a Multi-Objective calibration against itself when selecting one of three fitness functions in a Single-Objective GA. Ratios below the unity mean that the fitness function in a Single-Objective calibration achieved greater errors than itself in the Multi-Objective optimization. **C. Comparison to elitist GA.** The figure shows the ratio between the fractional errors to compare the *inverse* and the *elitist* strategies. Ratios below the unity mean that the selection and mutation implemented in Strategy3 achieved a lower error reduction compared to the initial population than the *elitist* strategy to select parents.

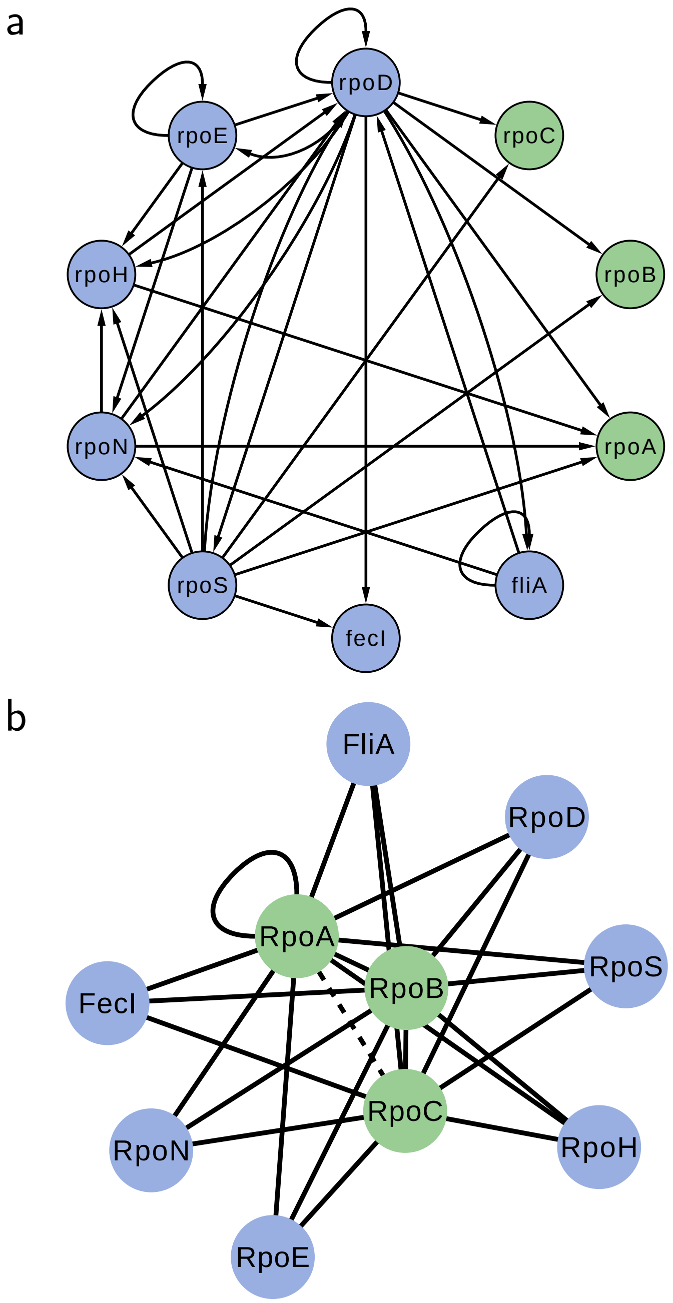

Figure S8: Biological networks employed to develop the Core Gene Regulatory Network Model. **A:** The Gene Regulatory Network is a directed network composed by 30 positive regulations and 10 genes, seven of which are sigma factors (light blue) and the remaining enconde the α, β, and β' subunits of the RNA Polymerase Core Enzyme (RpoA, B, and C). **B:** The Protein-Protein Interaction Network encoding the physical interactions between all proteins. The core enzyme is a tetramer composed of two α subunits, and one of each β and β' subunits (green nodes). The RNAP holoenzyme is completed with one of the seven sigma factors. The hard lines are physical interactions while the dashed line between RpoA and RpoC proteins denotes a indirect interaction.

Table S1. Inference of parameter uncertainty throughout one-leave-out Jackknife for the core GRN model. "Jack" refers to the Jackknife estimator corrected after bias consideration. "Mean" refers to the average of all subsamples, "SE" the standard error of subsamples, and "bias" refers to the Jackknife bias.

docking_rnap54_p1rpoa_rvs	jack: 0.548096	mean: 0.501163	SE: 0.440610	bias: -0.031289
docking_rnap38_p1rpoa_fwd	jack: 2.079586	mean: 0.357589	SE: 0.557333	bias: -1.147998
docking_rnap38_p1rpoa_rvs	jack: -0.278159	mean: 0.730746	SE: 0.223807	bias: 0.672603
docking_rnap70_p1rpob_fwd	jack: 0.306648	mean: 0.428711	SE: 0.268280	bias: 0.081375
docking_rnap70_p1rpob_rvs	jack: 1.211016	mean: 0.453764	SE: 0.205415	bias: -0.504834
docking_rnap38_p1rpob_fwd	jack: 0.283344	mean: 0.386651	SE: 0.358360	bias: 0.068871
docking_rnap38_p1rpob_rvs	jack: -0.333834	mean: 0.794340	SE: 0.286838	bias: 0.752116
docking_rnap70_p1rpod_fwd	jack: -0.924408	mean: 0.593744	SE: 0.485754	bias: 1.012101
docking_rnap70_p1rpod_rvs	jack: 1.178694	mean: 0.126788	SE: 0.037080	bias: -0.701271
docking_rnap24_p1rpod_fwd	jack: 1.445771	mean: 0.582415	SE: 0.288675	bias: -0.575570
docking_rnap24_p1rpod_rvs	jack: 0.173118	mean: 0.530660	SE: 0.253974	bias: 0.238362
docking_rnap32_p1rpod_fwd	jack: 0.669233	mean: 0.350031	SE: 0.411611	bias: -0.212802
docking_rnap32_p1rpod_rvs	jack: 1.391538	mean: 0.453387	SE: 0.259699	bias: -0.625433
docking_rnap28_p1rpod_fwd	jack: 0.199553	mean: 0.895794	SE: 0.135881	bias: 0.464161
docking_rnap28_p1rpod_rvs	jack: -0.194685	mean: 0.175885	SE: 0.175418	bias: 0.247047
docking_rnap54_p1rpod_fwd	jack: 1.403161	mean: 0.371097	SE: 0.334056	bias: -0.688043
docking_rnap54_p1rpod_rvs	jack: 0.379299	mean: 0.581213	SE: 0.362477	bias: 0.134609
rpoa_rpoa_rpob_rpoc_rpod_rvs	jack: 22.956947	mean: 47.709907	SE: 43.905436	bias: 16.501973
docking_rnap38_p1rpod_fwd	jack: -0.027449	mean: 0.675240	SE: 0.397401	bias: 0.468459
docking_rnap38_p1rpod_rvs	jack: -0.174941	mean: 0.268199	SE: 0.278194	bias: 0.295427
rpoa_rpoa_rpob_rpoc_rpoe_fwd	jack: -103.890327	mean: 53.425700	SE: 10.765071	bias: 104.877351
docking_rnap70_p1rpoe_fwd	jack: -0.056737	mean: 0.142428	SE: 0.141740	bias: 0.132776
docking_rnap70_p1rpoe_rvs	jack: -0.779746	mean: 0.613189	SE: 0.305522	bias: 0.928623
docking_rnap24_p1rpoe_fwd	jack: -0.575889	mean: 0.315666	SE: 0.527432	bias: 0.594370
docking_rnap24_p1rpoe_rvs	jack: 0.696355	mean: 0.540360	SE: 0.456712	bias: -0.103997
docking_rnap38_p1rpoe_fwd	jack: -0.145342	mean: 0.659037	SE: 0.630924	bias: 0.536253
docking_rnap38_p1rpoe_rvs	jack: 0.047703	mean: 0.529356	SE: 0.173448	bias: 0.321102
rpoa_rpoa_rpob_rpoc_rpoh_fwd	jack: -43.590670	mean: 68.995820	SE: 24.764705	bias: 75.057660
degrade_RNA_rpoHRBSRNA_rpoHCDS_k	jack: 0.102653	mean: 0.092558	SE: 0.011236	bias: -0.006730
degrade_RNA_rpoNRBSRNA_rpoNCDS_k	jack: 0.097976	mean: 0.085013	SE: 0.010174	bias: -0.008642
docking_rnap70_p1rpoh_fwd	jack: -1.535984	mean: 0.775170	SE: 0.123273	bias: 1.540769
docking_rnap70_p1rpoh_rvs	jack: -0.492714	mean: 0.583872	SE: 0.159728	bias: 0.717723
rpoa_rpoa_rpob_rpoc_rpoh_rvs	jack: 4.132941	mean: 54.259279	SE: 54.254576	bias: 33.417559
docking_rnap24_p1rpoh_fwd	jack: -1.062509	mean: 0.605495	SE: 0.584072	bias: 1.112003
docking_rnap24_p1rpoh_rvs	jack: -0.753176	mean: 0.594018	SE: 0.546206	bias: 0.898129

docking_rnap54_p1rpoh_fwd	jack: -0.270295	mean: 0.265867	SE: 0.207013	bias: 0.357441
docking_rnap54_p1rpoh_rvs	jack: -0.610393	mean: 0.358586	SE: 0.537333	bias: 0.645986
docking_rnap38_p1rpoh_fwd	jack: -0.829795	mean: 0.506609	SE: 0.235179	bias: 0.890936
docking_rnap38_p1rpoh_rvs	jack: 0.555719	mean: 0.434632	SE: 0.329177	bias: -0.080724
docking_rnap70_p1rpon_fwd	jack: 0.297563	mean: 0.804841	SE: 0.138248	bias: 0.338186
docking_rnap70_p1rpon_rvs	jack: -0.853719	mean: 0.591613	SE: 0.439740	bias: 0.963555
docking_rnap24_p1rpon_fwd	jack: -0.436682	mean: 0.602523	SE: 0.410908	bias: 0.692804
docking_rnap24_p1rpon_rvs	jack: 1.569712	mean: 0.600113	SE: 0.206707	bias: -0.646399
docking_rnap28_p1rpon_fwd	jack: -1.043139	mean: 0.693260	SE: 0.471540	bias: 1.157600
docking_rnap28_p1rpon_rvs	jack: 1.218488	mean: 0.560888	SE: 0.488593	bias: -0.438400
docking_rnap38_p1rpon_fwd	jack: 1.569730	mean: 0.343754	SE: 0.292290	bias: -0.817318
docking_rnap38_p1rpon_rvs	jack: 1.575125	mean: 0.458534	SE: 0.199591	bias: -0.744394
docking_rnap70_p1rpos_fwd	jack: 0.091168	mean: 0.452659	SE: 0.444664	bias: 0.240994
docking_rnap70_p1rpos_rvs	jack: -0.027586	mean: 0.442720	SE: 0.477901	bias: 0.313537
docking_rnap70_p1feci_fwd	jack: -0.395923	mean: 0.368121	SE: 0.338683	bias: 0.509362
docking_rnap70_p1feci_rvs	jack: 0.883405	mean: 0.638015	SE: 0.216305	bias: -0.163593
docking_rnap38_p1feci_fwd	jack: 1.945819	mean: 0.277128	SE: 0.249491	bias: -1.112461
docking_rnap38_p1feci_rvs	jack: 0.352451	mean: 0.508967	SE: 0.466181	bias: 0.104344
docking_rnap70_p1flia_fwd	jack: 1.616003	mean: 0.455350	SE: 0.254541	bias: -0.773769
docking_rnap70_p1flia_rvs	jack: -0.107507	mean: 0.287335	SE: 0.216224	bias: 0.263228
docking_rnap28_p1flia_fwd	jack: -0.366718	mean: 0.659787	SE: 0.132764	bias: 0.684337
docking_rnap28_p1flia_rvs	jack: 0.070267	mean: 0.347218	SE: 0.313470	bias: 0.184634
degrade_RNA_rpoARBSRNA_rpoACDS_k	jack: 0.131001	mean: 0.082656	SE: 0.026083	bias: -0.032230
rpoa_rpoa_rpob_rpoc_rpod_fwd	jack: -11.653844	mean: 8.633392	SE: 11.530999	bias: 13.524824
degrade_RNA_rpoBRBSRNA_rpoBCDSRNA_rpoCRBSRNA_r...	jack: 0.071978	mean: 0.081669	SE: 0.013136	bias: 0.006461
degrade_RNA_rpoERBSRNA_rpoECDS_k	jack: 0.113551	mean: 0.084511	SE: 0.009664	bias: -0.019360
rpoa_rpoa_rpob_rpoc_rpoe_rvs	jack: -47.122202	mean: 30.033222	SE: 32.353123	bias: 51.436949
degrade_RNA_rpoDRBSRNA_rpoDCDS_k	jack: 0.127786	mean: 0.079709	SE: 0.026003	bias: -0.032052
degrade_RNA_rpoSRBSRNA_rpoSCDS_k	jack: 0.108318	mean: 0.067690	SE: 0.026675	bias: -0.027086
degrade_RNA_fecIRBSRNA_fecICDS_k	jack: -0.033591	mean: 0.075229	SE: 0.015320	bias: 0.072546
rpoa_rpoa_rpob_rpoc_flia_rvs	jack: 199.318554	mean: 42.132678	SE: 48.424030	bias: -104.790584
degrade_RNA_fliARBSRNA_fliACDS_k	jack: 0.074741	mean: 0.053473	SE: 0.004697	bias: -0.014179
rpoa_rpoa_rpob_rpoc_flia_fwd	jack: -7.860493	mean: 56.354497	SE: 43.376035	bias: 42.809993
rpoa_rpoa_rpob_rpoc_rpos_fwd	jack: -25.413204	mean: 14.100088	SE: 24.370868	bias: 26.342195
rpoa_rpoa_rpob_rpoc_rpon_fwd	jack: 189.114757	mean: 31.464597	SE: 37.852692	bias: -105.100107
rpoa_rpoa_rpob_rpoc_feci_fwd	jack: 197.161354	mean: 46.756033	SE: 52.639915	bias: -100.270214
rpoa_rpoa_rpob_rpoc_feci_rvs	jack: 111.224186	mean: 40.733807	SE: 46.774729	bias: -46.993586
rpoa_rpoa_rpob_rpoc_rpos_rvs	jack: 17.207583	mean: 31.137103	SE: 14.568058	bias: 9.286347
docking_rnap70_p1rpoa_rvs	jack: 0.597349	mean: 0.686827	SE: 0.366964	bias: 0.059652

rpoa_rpoa_rpob_rpoc_rpon_rvs	jack: 153.220170	mean: 58.586580	SE: 35.334772	bias: -63.089060
docking_rnap32_p1rpoa_fwd	jack: 1.267096	mean: 0.363877	SE: 0.483783	bias: -0.602146
docking_rnap32_p1rpoa_rvs	jack: 2.104009	mean: 0.329714	SE: 0.389054	bias: -1.182864
docking_rnap70_p1rpoa_fwd	jack: 0.632337	mean: 0.345819	SE: 0.425477	bias: -0.191011
docking_rnap54_p1rpoa_fwd	jack: -0.502339	mean: 0.331603	SE: 0.343427	bias: 0.555961

Table S2. Inference of parameter uncertainty throughout 20 bootstrapping runs. The procedure allow a confidence interval of 90.0%

degrade_RNA_fecIRBSRNA_fecICDS_k	0.015418	0.093511
degrade_RNA_fliARBSRNA_fliACDS_k	0.011663	0.074076
degrade_RNA_rpoARBSRNA_rpoACDS_k	0.010000	0.094953
degrade_RNA_rpoBRBSRNA_rpoBCDSRNA_rpoCRBSRNA_rpoCCDS_k	0.029113	0.098912
degrade_RNA_rpoDRBSRNA_rpoDCDS_k	0.038780	0.096884
degrade_RNA_rpoERBSRNA_rpoECDS_k	0.031806	0.097227
degrade_RNA_rpoHRBSRNA_rpoHCDS_k	0.063836	0.099330
degrade_RNA_rpoNRBSRNA_rpoNCDS_k	0.020770	0.097391
degrade_RNA_rpoSRBSRNA_rpoSCDS_k	0.008518	0.091329
docking_rnap24_p1rpod_fwd	0.095833	0.942509
docking_rnap24_p1rpod_rvs	0.103773	0.993867
docking_rnap24_p1rpoe_fwd	0.010940	0.881966
docking_rnap24_p1rpoe_rvs	0.060300	0.906298
docking_rnap24_p1rpoh_fwd	0.037422	0.943149
docking_rnap24_p1rpoh_rvs	0.018008	0.829740
docking_rnap24_p1rpon_fwd	0.093583	0.916892
docking_rnap24_p1rpon_rvs	0.060983	0.847561
docking_rnap28_p1flia_fwd	0.059870	0.895893
docking_rnap28_p1flia_rvs	0.009676	0.924858
docking_rnap28_p1rpod_fwd	0.019887	0.961636
docking_rnap28_p1rpod_rvs	0.339933	0.919450
docking_rnap28_p1rpon_fwd	0.015624	0.844661
docking_rnap28_p1rpon_rvs	0.138558	0.904499
docking_rnap32_p1rpoa_fwd	0.019595	0.827524
docking_rnap32_p1rpoa_rvs	0.217859	0.956201
docking_rnap32_p1rpod_fwd	0.021209	0.835443
docking_rnap32_p1rpod_rvs	0.049484	0.920967
docking_rnap38_p1feci_fwd	0.017611	0.915298
docking_rnap38_p1feci_rvs	0.051926	0.948303
docking_rnap38_p1rpoa_fwd	0.009059	0.892295
docking_rnap38_p1rpoa_rvs	0.037370	0.961912
docking_rnap38_p1rpob_fwd	0.132154	0.956783
docking_rnap38_p1rpob_rvs	0.215169	0.993703
docking_rnap38_p1rpod_fwd	0.088594	0.976686
docking_rnap38_p1rpod_rvs	0.150762	0.981178
docking_rnap38_p1rpoe_fwd	0.050735	0.894490
docking_rnap38_p1rpoe_rvs	0.018819	0.940980
docking_rnap38_p1rpoh_fwd	0.055780	0.975849
docking_rnap38_p1rpoh_rvs	0.241838	0.966549
docking_rnap38_p1rpon_fwd	0.257689	0.968392
docking_rnap38_p1rpon_rvs	0.027071	0.892891
docking_rnap54_p1rpoa_fwd	0.018159	0.745950
docking_rnap54_p1rpoa_rvs	0.038679	0.866578
docking_rnap54_p1rpod_fwd	0.035440	0.848812
docking_rnap54_p1rpod_rvs	0.176343	0.962994
docking_rnap54_p1rpoh_fwd	0.006197	0.775382
docking_rnap54_p1rpoh_rvs	0.090734	0.974517
docking_rnap70_p1feci_fwd	0.019493	0.928720
docking_rnap70_p1feci_rvs	0.180631	0.896933
docking_rnap70_p1flia_fwd	0.130893	0.966198
docking_rnap70_p1flia_rvs	0.144216	0.987815

docking_rnap70_p1rpoa_fwd	0.016978	0.972855
docking_rnap70_p1rpoa_rvs	0.301900	0.952864
docking_rnap70_p1rpob_fwd	0.048642	0.780183
docking_rnap70_p1rpob_rvs	0.055855	0.941712
docking_rnap70_p1rpod_fwd	0.202404	0.965757
docking_rnap70_p1rpod_rvs	0.175459	0.819108
docking_rnap70_p1rpoe_fwd	0.042815	0.931646
docking_rnap70_p1rpoe_rvs	0.046631	0.858377
docking_rnap70_p1rpoh_fwd	0.014443	0.940546
docking_rnap70_p1rpoh_rvs	0.087262	0.857371
docking_rnap70_p1rpon_fwd	0.390051	0.951194
docking_rnap70_p1rpon_rvs	0.188165	0.935545
docking_rnap70_p1rpos_fwd	0.118926	0.978016
docking_rnap70_p1rpos_rvs	0.073125	0.947389
rpoa_rpoa_rpob_rpoc_feci_fwd	8.337535	95.107190
rpoa_rpoa_rpob_rpoc_feci_rvs	1.715186	71.856660
rpoa_rpoa_rpob_rpoc_flia_fwd	22.552620	87.546220
rpoa_rpoa_rpob_rpoc_flia_rvs	1.433090	99.534650
rpoa_rpoa_rpob_rpoc_rpod_fwd	0.070744	81.594050
rpoa_rpoa_rpob_rpoc_rpod_rvs	28.688300	94.725920
rpoa_rpoa_rpob_rpoc_rpoe_fwd	1.552307	88.211000
rpoa_rpoa_rpob_rpoc_rpoe_rvs	0.470571	93.895510
rpoa_rpoa_rpob_rpoc_rpoh_fwd	6.008725	96.796830
rpoa_rpoa_rpob_rpoc_rpoh_rvs	1.057004	98.138970
rpoa_rpoa_rpob_rpoc_rpon_fwd	3.466332	77.086490
rpoa_rpoa_rpob_rpoc_rpon_rvs	7.007182	75.743280
rpoa_rpoa_rpob_rpoc_rpos_fwd	0.210678	78.488350
rpoa_rpoa_rpob_rpoc_rpos_rvs	29.672810	90.964620
